# Fluorescence Lifetime Imaging of Alterations to Cellular Metabolism by Domain 2 of the Hepatitis C Virus Core Protein

**DOI:** 10.1371/journal.pone.0066738

**Published:** 2013-06-24

**Authors:** Nirmal Mazumder, Rodney K. Lyn, Ragunath Singaravelu, Andrew Ridsdale, Douglas J. Moffatt, Chih-Wei Hu, Han-Ruei Tsai, John McLauchlan, Albert Stolow, Fu-Jen Kao, John Paul Pezacki

**Affiliations:** 1 Institute of Biophotonics, National Yang-Ming University, Taipei, Taiwan; 2 National Research Council of Canada, Ottawa, Ontario, Canada; 3 Department of Chemistry, University of Ottawa, Ottawa, Ontario, Canada; 4 Department of Biochemistry, Microbiology and Immunology, University of Ottawa, Ottawa, Ontario, Canada; 5 Medical Research Council - University of Glasgow Center for Virus Research, Glasgow, United Kingdom; 6 Department of Physics, Queen’s University, Kingston, Ontario, Canada; University of North Carolina School of Medicine, United States of America

## Abstract

Hepatitis C virus (HCV) co-opts hepatic lipid pathways to facilitate its pathogenesis. The virus alters cellular lipid biosynthesis and trafficking, and causes an accumulation of lipid droplets (LDs) that gives rise to hepatic steatosis. Little is known about how these changes are controlled at the molecular level, and how they are related to the underlying metabolic states of the infected cell. The HCV core protein has previously been shown to independently induce alterations in hepatic lipid homeostasis. Herein, we demonstrate, using coherent anti-Stokes Raman scattering (CARS) microscopy, that expression of domain 2 of the HCV core protein (D2) fused to GFP is sufficient to induce an accumulation of larger lipid droplets (LDs) in the perinuclear region. Additionally, we performed fluorescence lifetime imaging of endogenous reduced nicotinamide adenine dinucleotides [NAD(P)H], a key coenzyme in cellular metabolic processes, to monitor changes in the cofactor’s abundance and conformational state in D2-GFP transfected cells. When expressed in Huh-7 human hepatoma cells, we observed that the D2-GFP induced accumulation of LDs correlated with an increase in total NAD(P)H fluorescence and an increase in the ratio of free to bound NAD(P)H. This is consistent with an approximate 10 fold increase in cellular NAD(P)H levels. Furthermore, the lifetimes of bound and free NAD(P)H were both significantly reduced – indicating viral protein-induced alterations in the cofactors’ binding and microenvironment. Interestingly, the D2-expressing cells showed a more diffuse localization of NAD(P)H fluorescence signal, consistent with an accumulation of the co-factor outside the mitochondria. These observations suggest that HCV causes a shift of metabolic control away from the use of the coenzyme in mitochondrial electron transport and towards glycolysis, lipid biosynthesis, and building of new biomass**.** Overall, our findings demonstrate that HCV induced alterations in hepatic metabolism is tightly linked to alterations in NAD(P)H functional states.

## Introduction

Hepatitis C virus (HCV) infection is a global health concern and a leading cause of hepatocellular carcinoma and liver transplantation [1]–[3]. The HCV RNA genome is a ∼9.6 kb RNA virus that encodes for a ∼3000 amino acid polyprotein that is cleaved into three structural proteins (core, E1 and E2) as well as seven nonstructural proteins (p7, NS2, NS3, NS4A/B, and NS5A/B) each playing important roles in the HCV life cycle [4]. The virus is highly heterogeneous, with genotypic variants of HCV differing in their responses to clinical interventions [5]. Although effective treatments are available against HCV, these treatments still exhibit side effects and possess varying efficacy against some subpopulations of patients [5], [6]. Given the relatively small size of its genome, HCV relies on hijacking host factors and pathways to facilitate its viral life cycle [7], [8].

Hepatic lipid metabolism represents one pathway that is intimately linked to HCV pathogenesis [7], [9]. In fact, HCV infection is associated with steatosis in over 40% of patients [10]. This hepatic lipid accumulation results from virus-induced alterations in host lipid homeostasis [7], [9]. Specifically, the virus promotes lipid synthesis and inhibits lipid secretion and catabolism which lead to formation of lipid droplets (LDs) and lipid-rich membranous webs. These intracellular structures are crucial for replication and assembly of the virus [7], [9]. In addition to its roles in viral capsid formation and packaging of viral RNA [11], the HCV core protein is known to play a key role in virus induced changes in host metabolic flux. The core protein strongly associates with LDs and is progressively loaded onto LDs over time after HCV infection is established [12]. This association is directly correlated with the ability of HCV to produce infectious particles [12]–[15]. HCV core protein also controls the size, subcellular localization, and movement of LDs on microtubules [13], [16]. These core-induced changes to cytoplasmic LDs represent a subset of the changes HCV core induces in host metabolism to create an environment that is advantageous to the virus.

Additionally, HCV core protein expression independently induces shifts in metabolic flux which have a net effect of increasing cellular lipid content. This involves up-regulation of *de novo* lipid synthesis, suppression of fatty-acid β-oxidation, and impairment of lipoprotein-coupled lipid efflux [17]. HCV core protein stimulates fatty acid synthesis [18], [19], induces LD biogenesis [20], and decreases LD turnover [21]. The severity of these effects varies by HCV genotype. Specifically, the HCV core protein from genotype 3a has shown the most pronounced effects that have been directly implicated with steatosis [22]. The mature form of HCV core protein comprises of two domains. The second domain (D2) contains two α-helices that are hydrophobic and enable anchoring to the endoplasmic reticulum (ER) and a strong association with cytoplasmic LDs [23]. D2 has been shown to independently alter the composition of proteins bound to the LD and changes LD localization and trafficking [24]. It remains unclear whether D2 is similarly sufficient to induce the alterations in metabolic flux observed during HCV core expression.

Two-photon fluorescence lifetime imaging microscopy (TP-FLIM) represents a non-invasive imaging technique to visualize alterations in metabolic state, by tracking intrinsic fluorophores present in the cell, such as nicotinamide adenine dinucleotide (NADH) and its phosphorylated form (NADPH). NAD(P)H is a key coenzyme in glycolysis and oxidative energy metabolism that acts as a principal electron carrier in energy transduction and biosynthetic processes [25]. NADH plays a key role in catalyzing catabolic reactions in the mitochondria and cytosol while NADPH is a cofactor in anabolic reactions and plays a key role as a cellular antioxidant [25]. An imbalance of these co-factors can provide evidence for differential energy flow. Although, the coenzyme exists in an oxidized [NAD(P)+] and a reduced [NAD(P)H] form, only the reduced form is intrinsically fluorescent [26]. With two photon excitation at 730–750 nm wavelengths, the cellular intrinsic fluorescence is dominated by NAD(P)H species [27]. Careful selection of wavelengths for excitation and emission can enable non-destructive detection with spatial and temporal resolution [28].

The fluorescent lifetime represents the time a molecule spends in its excited state before returning to its ground state. Observations of the changes in NAD(P)H fluorescence lifetime give additional insight into the cofactor’s microenvironment, including its conformation, interactions with other molecules in the system, and changes in pH [28], [29]. Subpopulations of NAD(P)H exhibit distinct lifetimes corresponding to the free and protein bound forms of NAD(P)H [28]. Assuming a model fitting bi-exponential fluorescence decay enables monitoring of the ratio of free to protein bound NAD(P)H. The relative quantities of the free and bound forms of this coenzyme can give insight into the metabolic state of the cell as the functional roles of the cofactors are dependent on the proteins with which they interact. The mean lifetime of protein bound NAD(P)H (τ_2_) is ∼2.3–3.0 ns, while the free form has a short lifetime (τ_1_) of ∼0.3–0.4 ns [28]. Measurements of the fluorescence intensity and lifetime of NAD(P)H provides a unique visualization of cellular metabolic signatures.

FLIM imaging has been applied widely to the visualization of changes in metabolism and energy consumption in mammalian cells. Previous studies have utilized NAD(P)H FLIM to demonstrate changes in metabolism in stem cells and cancer cells, both *in vitro* [27], [30]–[32] and *in vivo* [33]. With regards to host-pathogen interactions, autofluorescence lifetime analysis has been utilized to uncover the alterations in metabolic flux induced by bacterial infections [34], [35]. Previous work analyzing HeLa cell metabolism in the presence of enterohemorrhagic *Escherichia coli* revealed an increase in the relative concentration of free NADH, which suggested a decrease in oxidative phosphorylation over the course of infection [34]. Furthermore, FLIM imaging of NAD(P)H has been used to study the metabolism of the bacterium *Chlamydia trachomatis* and its interactions, effects, and crosstalk with its host cell during infection [35]. Szaszak *et al.* analyzed host and pathogen metabolic changes during intracellular *C. trachomatis* infections and demonstrated a tight link between changes in host cell metabolism and chlamydial development [35]. The authors directly linked changes in chlamydial growth and progeny, induced by glucose starvation and interferon treatment, with significant changes of the NAD(P)H fluorescence lifetimes inside bacterial inclusions. These studies suggested that FLIM imaging of NAD(P)H during host-virus interactions could also potentially shed light on changes to cellular metabolism induced by viral proteins.

Herein, we use a multimodal imaging approach, employing both coherent anti-Stokes Raman scattering (CARS) microscopy and FLIM, to establish metabolic signatures for hepatoma cells in the presence and absence of HCV core D2 expression. CARS imaging revealed that D2 expression was sufficient to induce hepatic lipid accumulation and an increase in LD size and number. We also observed an increase in NAD(P)H autofluorescence, which correlates to an approximate 10 fold increase in reduced NADH levels. TP-FLIM imaging revealed that HCV core D2’s influence on energy flow is directly correlated with a decrease in lifetimes of both free and bound NAD(P)H as well as an overall increase in free NAD(P)H levels, which we partially attribute to D2-induced disruption of mitochondrial electron transport. These results demonstrate that FLIM is a powerful tool for probing host-cell metabolic changes in the presence of HCV.

## Results

### Expression of HCV core D2 is Sufficient to Cause an Increase in Lipid Droplet Size and Number

Previous studies have clearly demonstrated that HCV core expression independently induces alterations in hepatic metabolic flux [10], [11], [13], [23]. As a strategy to investigate HCV core dynamics within living hepatocytes, Shavinskaya *et al.* incorporated a fusion DNA plasmid that fuses D2 of core protein to a green fluorescent protein (GFP) at its N-terminus [14]. The GFP-tagged D2 of core is able to associate with the LD surface without GFP hindering D2’s binding to the LD surface. We wished to apply this construct to investigate whether D2 expression can independently induce alterations in metabolic flux observed during HCV infection [36], [37], [38].

**Figure 1:**
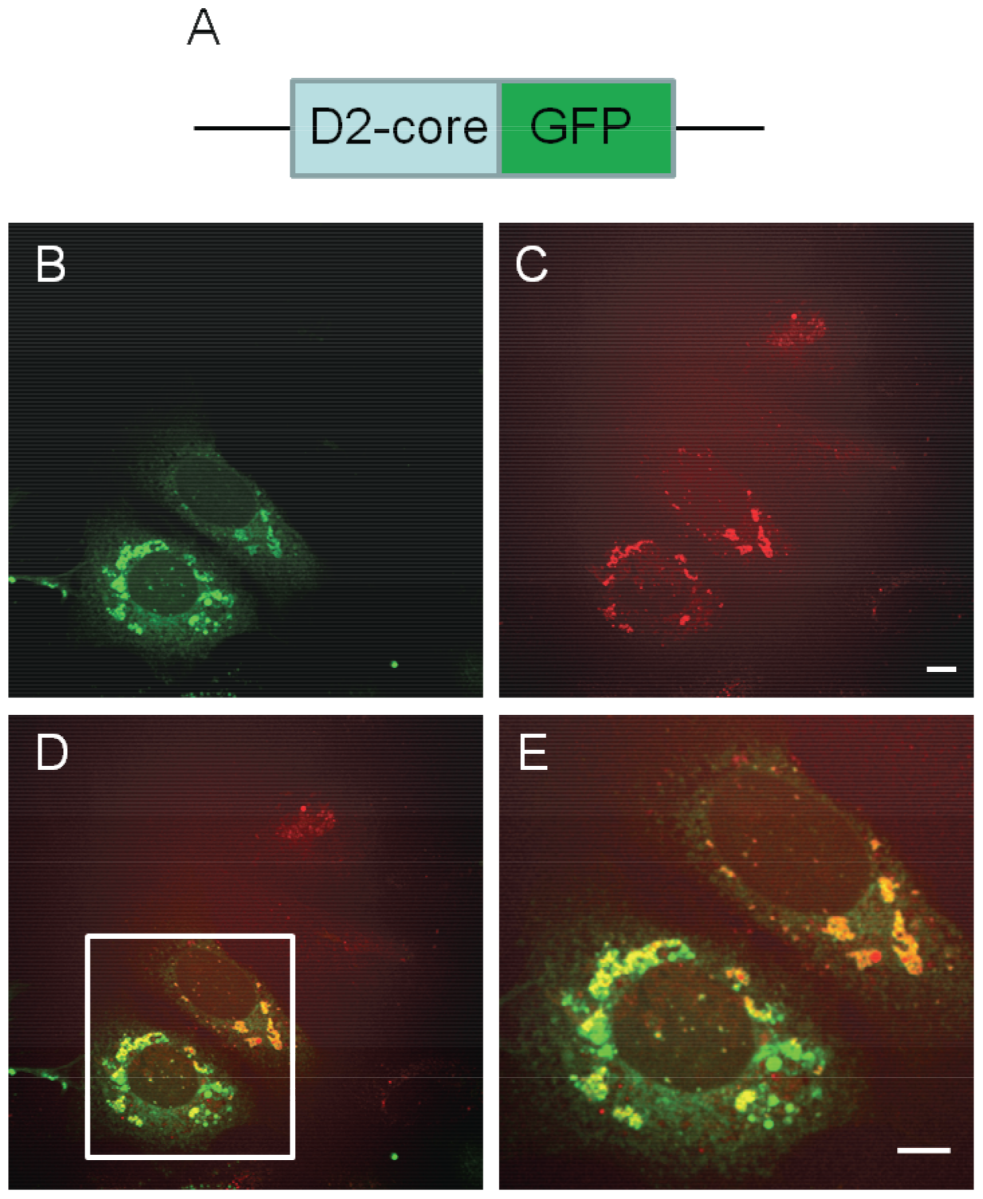
Domain 2 of HCV core protein induces lipid accumulation in Huh7 cells. Huh7 cells were fixed and imaged by CARS and TPF microscopy 24 hours post-transfection with D2-GFP fusion protein. (A) Diagram of the HCV D2-GFP fusion protein. (B)–(E) Simultaneous CARS and two-photon fluorescence image of Huh7 cells expressing D2-GFP. (B) Typical two-photon fluorescence image showing localization of D2-GFP (green); (C) CARS image of lipid droplets (red) in the same field of view; (D) Overlay of the CARS and two-photon fluorescence images; (E) Magnified image from the inset of (D). Representative images are shown of three biological replicates. Scale bar = 10 µm.

CARS microscopy is a convenient live-cell imaging tool for monitoring changes in LD localization, size, and abundance in living cells [16], [39]–[44] and has previously been successfully applied to the study of HCV host-virus interactions [16], [41], [42], [44]. Here we used simultaneous two-photon fluorescence (TPF) and CARS microscopy to track D2-GFP localization and measure changes in the hepatic lipid content of Huh7 cells. The cells were transfected with the vector expressing D2-GFP fusion protein, and fixed 24 hours post-transfection. Simultaneous CARS and TPF revealed an increase in LD size and number in cells transfected with the D2-GFP construct (Figure 1, Figure S1) compared to adjacent non-transfected cells. As an additional control, cells expressing GFP alone displayed no changes in LD morphology (Figure S2). The changes to LD size occurred rapidly and in conjunction with the appearance of the signal associated with D2-GFP fusion protein - typically within 8 h of transfection of the plasmid expressing the core protein. Similar to full-length core, the core D2 fusion protein localized around LDs and induced recruitment of LDs to the perinuclear region – consistent with previous reports [14]. Previous studies have revealed that HCV core down-regulates the expression of PPAR-α, a transcription factor which activates the expression of genes associated with fatty acid catabolism [45]–[48]. Quantitative RT-PCR analysis of D2-GFP transfected Huh7 cells revealed a consistent 30% decrease in PPAR-α gene expression relative to mock (Figure S3). These results indicate that expression of domain 2 of the core protein is sufficient to give rise to metabolic changes in the host cell.

### D2-GFP Expression Alters NAD(P)H Abundance and Localization

**Figure 2:**
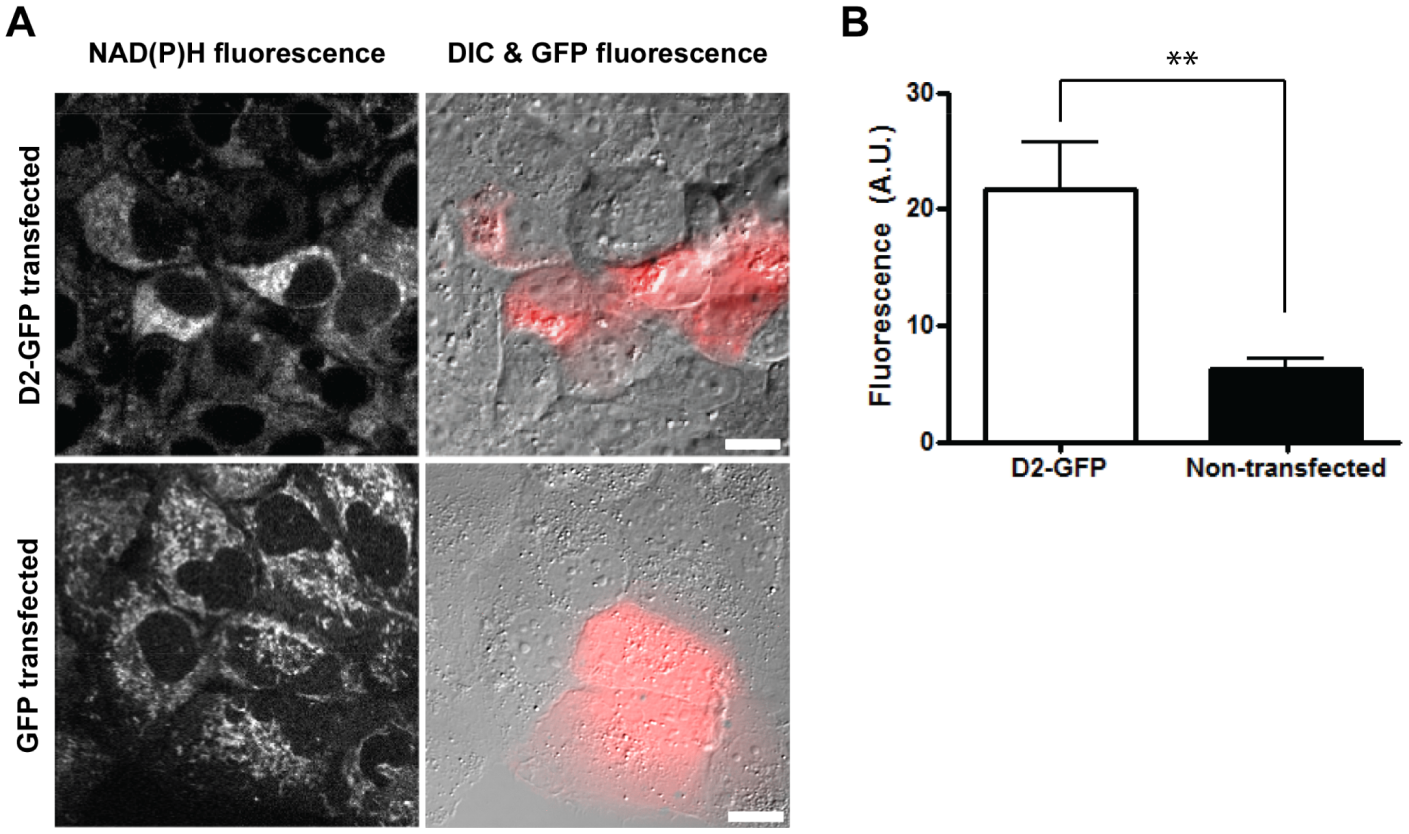
Domain 2 of HCV core protein increases NAD(P)H fluorescence. Huh7 cells were transfected with plasmids encoding D2-GFP or GFP. The cells were fixed and imaged 24 hours post-transfection with TPF for NAD(P)H fluorescence, DIC, and CCD camera for GFP fluorescence. (A) Left panels: Grayscale images of NAD(P)H fluorescence intensity signals. Right panels: Overlay of DIC images and GFP fluorescence (red). Representative images are shown from two biological replicates. Scale bar = 10 µm. (B) Quantitative analysis of NAD(P)H fluorescence intensity in D2-GFP transfected cells and neighbouring non-transfected cells. Error bars represent standard deviation (n≥3; **p<0.01).

We sought to examine D2-GFP expression’s influence on NAD(P)H levels and localization. Transfected cells were identified by imaging with standard wide-field fluorescence. Fields with fluorescent cells were identified using GFP optimized excitation and emission wavelengths. TPF imaging, with excitation centered at 740 nm and the emission filter centered around 450 nm, enabled visualization of NAD(P)H autofluorescence while rejecting GFP fluorescence. Huh7 cells transfected with D2-GFP possessed an approximately 3 fold increase in cellular autofluorescence compared to non-transfected cells in the same image field (Figure 2; Figure S4). Importantly, cells transfected with GFP alone showed no increase in NAD(P)H fluorescence (Figure 2). Interestingly, in non-transfected Huh7 cells, the majority of autofluorescence appeared to localize in distinct foci of the cell (Figure S5). Previous work suggests these observed foci correspond to mitochondria as the majority of NAD(P)H autofluorescence originates from mitochondria [49], where the coenzyme plays a critical role as an electron carrier for oxidative phosphorylation. In D2-GFP transfected cells, autofluorescence signal appeared to be more diffuse throughout the cell. This was further illustrated through calculation of the coefficients of variances in autofluorescence profiles from D2-GFP and GFP transfected cells (Figure S5C). We observed a decreased coefficient of variance for autofluorescence derived from D2-GFP transfected cells, indicating there was a more homogeneous spread of signal in the cytoplasm, as opposed to the distinct foci observed in the GFP transfected cells. Collectively, this data demonstrates that D2-GFP induces an increase in NAD(P)H levels, and alters the cellular distribution of the coenzyme. These observations are consistent with previous studies reporting an increase in NADH/NAD+ ratios in HCV infected cells [50], [51].

### TP-FLIM of D2-GFP Expressing Cells Reveals Changes in NAD(P)H Lifetime

**Figure 3:**
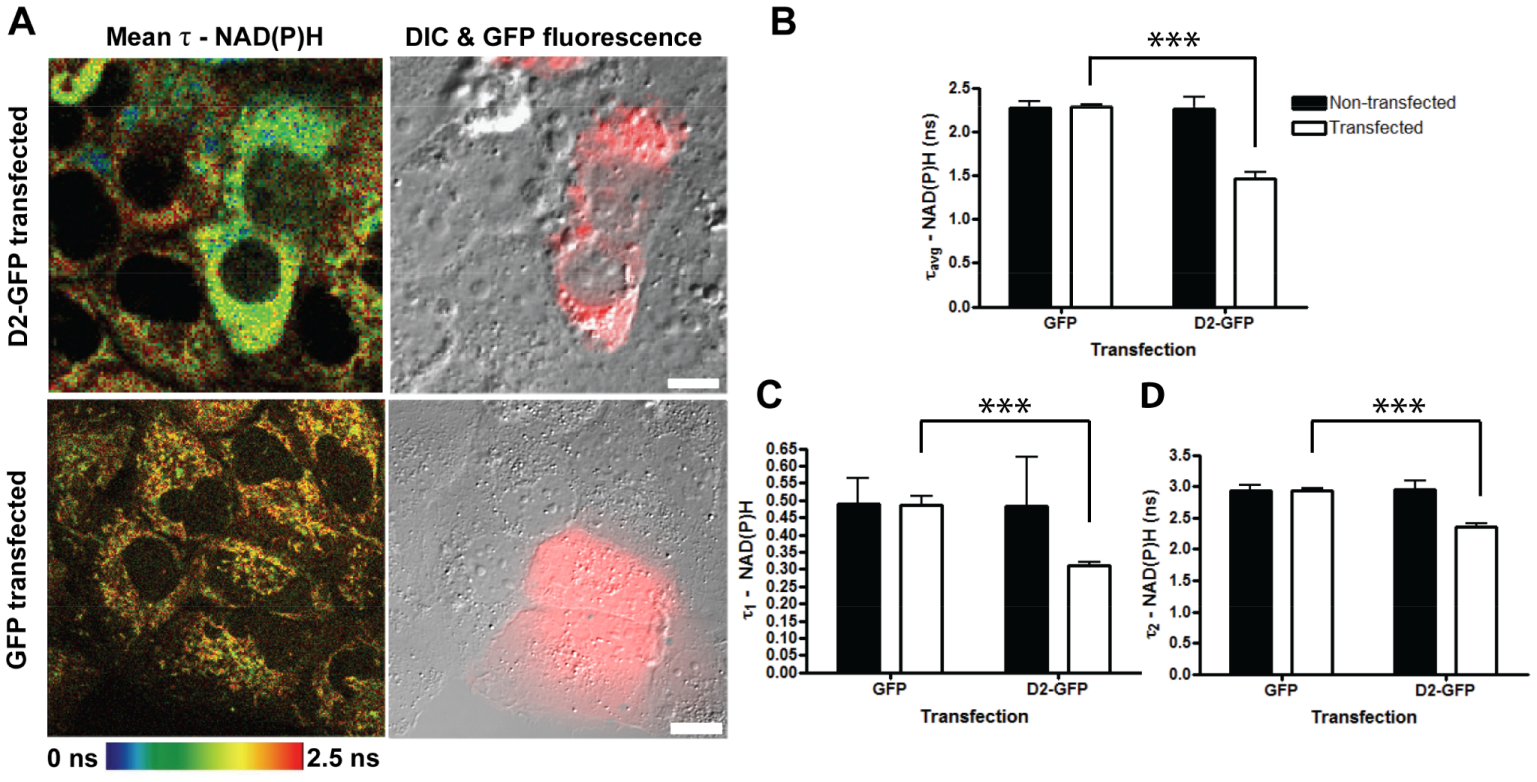
Domain 2 of HCV core induces alterations in NAD(P)H microenvironment. Huh7 cells were transfected with either D2-GFP or GFP. The cells were imaged 24 hours post-transfection with FLIM for NAD(P)H lifetimes, DIC, and CCD camera for GFP fluorescence. (A) Left panels: Pseudo-colored images of average NAD(P)H lifetimes. Right panels: Overlay of DIC images and GFP fluorescence (red). Representative images are shown of two biological replicates. Scale bar = 10 µm. (B) Quantitative analysis of NAD(P)H mean lifetimes. (C)–(D) Quantitative analysis of average lifetimes for (C) free and (D) bound NAD(P)H in GFP or D2-GFP expressing cells and non-transfected neighbouring cells. Error bars represent standard deviation (n≥12; ***p<0.001).

We then examined the effects of D2 on the NAD(P)H lifetimes to investigate D2’s effects on the coenzymes’ protein binding or microenvironment. Our TP-FLIM setup (Figure S6) produced a fluorescence lifetime decay curve, which was fitted with two exponential components (Figure S7). A good fit is characterized by a χ^2^ close to 1 and residuals showing no noticeable systematic variations. Both lifetimes (τ_1_ and τ_2_) and amplitudes (a_1_ and a_2_ - population sizes of molecules with the different decay rate) were obtained from fitting optimization software. The fits consistently produced lifetime values in the ranges of 0.2–0.4 ns and 2.0–3.0 ns for the free and bound forms of NAD(P)H, respectively. These values are similar to what has been previously reported [27]. Cells expressing D2 exhibited a significant decrease in NAD(P)H average lifetime (τ_avg_) from 2.3±0.3 ns to 1.5±0.3 ns (Figure 3A, B; Figure S8) (p<0.01). This decrease was consistently observed in comparison to either adjacent non-transfected cells or cells transfected with a plasmid expressing GFP alone. This suggested a specific alteration in NAD(P)H microenvironment in the presence of D2 expression. This model was further supported by the decrease in the fluorescence lifetimes of the free (τ_1_) and bound (τ_2_) form in the presence of D2 expression in Huh7 cells (Figure 3C, D). The lifetime of free NAD(P)H decreased from 0.49±0.9 ns to 0.31±0.3, while the lifetime of bound NAD(P)H decreased from 3.0±0.3 ns to 2.4±0.2 ns. These decreases were once again consistent in comparison to either non-transfected adjacent cells or cells transfected with GFP alone. The results indicate that D2 expression decreases NAD(P)H τ_1_ and τ_2_, which correlates with D2 induced modulations in cellular lipid metabolism.

### Increased Amount of Free NAD(P)H in Host Cells Expressing D2

**Figure 4:**
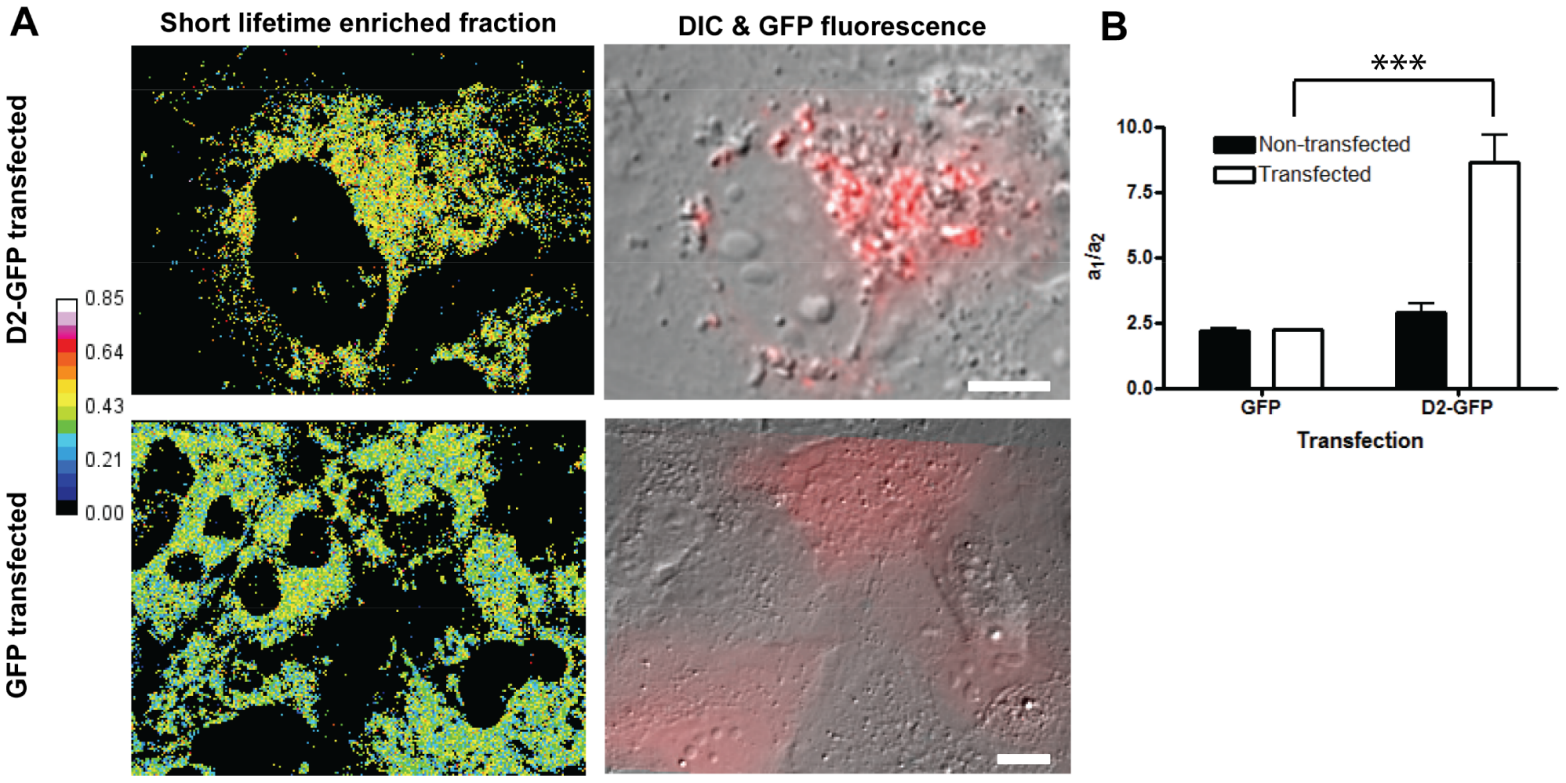
D2 expression induces enrichment for short lifetime forms of NAD(P)H. Huh7 *c*ells were transfected with either D2-GFP or GFP alone. Cells were imaged 24 hours post-transfection with FLIM for NAD(P)H lifetimes, DIC, and CCD camera for GFP fluorescence. (A) Overlay of DIC images and GFP fluorescence (red) are shown in right panels. Pseudo-colored images of the fraction of the fast decaying component (short lifetime) are shown in the left panels. The fraction was calculated as described in Materials and Methods. The range goes from 0 to 0.85 in evenly spaced increments on the colour scale. Pixels with less than 20 counts were set to 0. Scale bar = 10 µm. (B) Quantitative analysis of ratio of a_1_/a_2_ in Huh7 cells. ROIs (n≥12; ***p<0.001) were taken from two independent experiments. Error bars represent standard deviation.

Analysis of the amplitudes derived from the bi-exponential model (Figure 4) allows for the measurement of the ratio of free to protein-bound NAD(P)H (a_1_/a_2_). This ratio was increased over 3 fold (Figure 4B). This indicates that there is a much higher population of short lifetimes in transfected cells. Assuming that the changes in lifetime are dominated by differences in non-radiative processes, fluorescence lifetime is directly proportional to quantum yield [52], [53]. We can then estimate the magnitude of the D2-induced increase in NAD(P)H levels based on our empirically derived lifetimes, amplitudes and differences in NAD(P)H fluorescence intensity (refer to Text S1). Assuming negligible differences in two photon excitation cross-sections and refractive indices for the bound and unbound forms of NAD(P)H in D2 transfected and non-transfected samples, we estimate an approximate 10-fold increase in NAD(P)H levels. Collectively, our data demonstrates that D2 expression increases the abundance of NAD(P)H, and this is mainly attributed to an increase in the free NAD(P)H population.

## Discussion

HCV’s modulation of host lipid metabolism has been well documented [9], [54]. The virus hijacks hepatic lipid pathways to facilitate virtually every step of its life cycle. These alterations in host cellular lipid metabolism results in an increase in steady-state pools of *de novo* synthesized fatty acids and neutral lipids [37]. These lipids are used to modify ER membranes [9] and LDs [55] that are both utilized by HCV for replication, viral particle assembly, and secretion. Our results herein provide evidence that D2 of the HCV core protein can independently modulate hepatic metabolic flux.

Previous studies have demonstrated that HCV infection is accompanied by large shifts in metabolic fluxes [36]–[38], with over 10 fold increases in certain lipid metabolites [37]. This is consistent with hepatic lipid accumulation observed by CARS microscopy in the presence of HCV core D2-GFP expression (Figure 1). This steatotic phenotype represents a significant diversion of carbon sources into anabolic processes – similar to what is observed in highly proliferative cells. A metabolic shift toward increased biomass often correlates with an increase in NAD(P)H/NAD(P)+ ratio [56]. Consistent with this, previous reports correlate elevated NADH/NAD+ ratios with increased HCV replication [50], [51], [57]. In fact, a recent study elucidated that HCV core expression alone was sufficient to induce increased NADH/NAD+ [57]. These studies probing NADH/NAD+ ratios involved biochemical assays, which may inadvertently perturb the biological system. Imaging of NAD(P)H autofluorescence circumvents the issues associated with these traditional approaches, while allowing for visualization of the coenzyme’s subcellular localization. Our results demonstrate that expression of HCV core D2 upregulates NAD(P)H levels ∼10 fold – consistent with its ability to independently induce a steatotic phenotype. Yang *et al.* previously reported that an increase in NAD(P)H autofluorescence change precedes induction of oxidative stress in HepG2 cells treated with cadmium [58]. HCV core protein has previously been shown to induce the formation of reactive oxygen species (ROS) [59], a mechanism which has been linked to HCV-induced hepatic steatosis [60]. A similar sequential phenomena may result in D2’s induction of NAD(P)H accumulation. Overall, expression of HCV core’s D2 is sufficient to significantly modulate the hepatic lipid homeostasis and metabolism.

**Figure 5:**
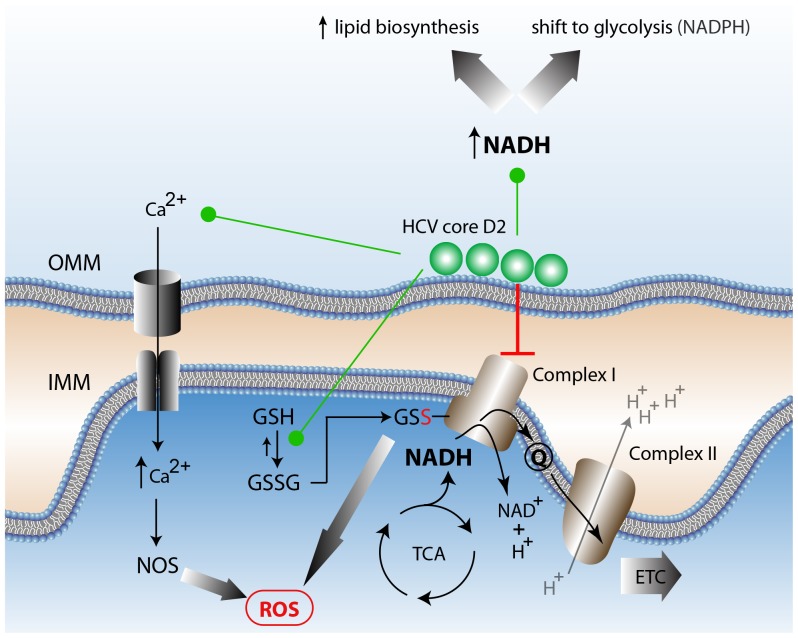
Proposed model of HCV core D2-induced alterations in metabolic homeostasis. The observed increase in NAD(P)H levels can be attributed to several plausible mechanisms [61]. HCV core D2 association with the outer mitochondrial membrane (OMM) modulates calcium ion (Ca^2+^) transporters’ activity. This interaction can elicit calcium ion influx into the mitochondrial matrix, which results in increased reactive oxygen species (ROS). Subsequent accumulation of oxidized glutathione (GSSG) and its association with Complex I can lead to disruption of the electron transport chain (ETC). D2 may also directly interaction with Complex I or NDUFS2 to inhibit Complex I activity, resulting in increased reduced NADH [63]. The increased NAD(P)H may allow for increased lipid biosynthesis or a shift to glycolysis within infected cells. This alteration in the cofactor’s protein binding and localization can account for changes in the NAD(P)H lifetimes observed.

Our results reveal a more diffuse NAD(P)H autofluorescence throughout the cytoplasm in HCV core D2-expressing cells (Figure S5). This is further demonstrated by the decreased variance for the autofluorescence signal observed in D2-GFP expressing cells compared to GFP expressing cells. This is consistent with observations in *C. trachomatis* infected cells, where increased non-mitochondrial NAD(P)H fluorescence was observed [35]. In our studies, the non-mitochondrial NAD(P)H fluorescence likely arises from D2-mediated disruption of mitochondrial bioenergetics, similar to what has previously been observed in the presence of HCV core expression (Figure 5) [61].

The D2-induced increase in cellular autofluorescence from two-photon excitation centered at 740 nm (Figure 2) originates from the metabolic coenzymes NADH and NADPH. NADH is the primary electron donor in oxidative phosphorylation and plays a key role in aerobic respiration [25]. Increased levels of NADH in the presence of HCV correlate with increased fatty acid synthesis and reduced fatty acid oxidation [62]. Interestingly, a yeast two hybrid screen by Tripathi *et al.* had elucidated a mitochondrial protein NADH dehydrogenase [ubiquinone] iron-sulfur protein 2 (NDUFS2), a key protein in the mitochondrial respiratory chain, as an HCV core interacting partner [63]. Korenaga *et al.* further demonstrated that electron transfer to ubiquinone is halted at complex I in oxidative phosphorylation, which prevents electron transport to subsequent complexes II-V [64]. As a consequence, NADH would be unable to transfer its electrons to complex I of the electron transport chain. Hence, D2-mediated disruption of the mitochondrial electron transport chain would similarly explain an HCV induced build-up of NADH levels (Figure 5).

Major sources of the phosphorylated form of NADH, NADPH, include the pentose phosphate pathway (PPP), malic enzyme, aldehyde dehydrogenase, and NADP-linked isocitrate dehydrogenase (IDH) [25]. The observed increase in cellular autofluorescence may also be attributed to an HCV core D2-induced increase in NADPH levels as this model would be consistent with previously observed increases in PPP intermediates in HCV-infected cells 24 hours post-HCV infection [38]. Diamond and co-workers observed proteome changes in HCV-infected cells consistent with a shift toward glycolysis, and the production of glutamine and lactate rather than shuttling intermediates into producing high amounts of energy in the form of ATP through oxidative phosphorylation [36]. These protein expression changes were highlighted by a marked increase in lactate dehydrogenase and NADP-independent isocitrate dehydrogenase (IDH2), both enzymes capable of regenerating NADPH levels. IDH2 also redirects α-ketoglutarate to citrate, thereby reversing TCA flux, and generating another source of acetyl-CoA for lipid synthesis – consistent with HCV core’s established induction of neutral lipid accumulation [16]. Overall, D2 induction of NAD(P)H levels would be in line with an HCV-induced shift to anabolism as the cofactor is used for both fatty acid elongation and cholesterol synthesis [25].

TP-FLIM allows for non-invasive determination of the NAD(P)H binding state as well as provides information on the cofactors’ microenvironment. Our results demonstrate a significant alteration in NAD(P)H microenvironment in the presence of D2 expression. Both the free and protein-bound lifetimes of NAD(P)H are significantly decreased in cells transfected with D2-GFP (Figure 3). This suggests an alteration in the microenvironment occurred for both populations of NAD(P)H. Considering that lipogenic enzymes are upregulated during HCV core overexpression, a change in NAD(P)H lifetime could result from the shuttling of NAD(P)H binding towards enzymes involved in these pathways. For example, fatty acid synthase (FASN) utilizes NADPH as a reducing equivalent for the reaction of acetyl-CoA and malonyl-CoA to produce palmitate, a major constituent of fatty acid biosynthesis [65]. HCV’s induction of FASN expression has been well documented, and, in order to facilitate increased lipogenesis, there would be a requirement for more cytosolic NADPH [18], [19]. Satisfying this viral requirement would account for the observed increased cytosolic localization and altered lifetimes of NAD(P)H in the presence of D2 expression. Additionally, our results indicate a three-fold shift in NAD(P)H from the bound to free form (Figure 4), similar to what was observed in precancerous hamster cheek epithelial cells when compared to normal epithelia [33]. Alternatively, the observed shift to free NAD(P)H may potentially result from the previously discussed core-induced disruption of mitochondrial electron transport [64].

In conclusion, FLIM has allowed us to independently assess and quantify NAD(P)H changes at the molecular level without perturbing the sensitive environment of these oxidation and reduction intermediates. We have shown that expression of HCV core D2 is sufficient to increase levels of the free form of NAD(P)H and alter the coenzyme’s microenvironment. These overall changes of metabolic co-enzymes are consistent with core’s proposed roles in up-regulating lipid biosynthesis and disruption of the mitochondrial electron transport chain. Our results suggest that D2 of the mature core protein independently induces changes in metabolic flux, consistent with a primary role for D1 in HCV RNA binding [66]–[68]. From our observations, D2 is sufficient to cause large changes in metabolic flux in the host liver cell and is a prime example of how a virus utilizes its genome efficiently to induce pro-viral alterations in host pathways. This work represents, to the best of our knowledge, the first application of fluorescence lifetime imaging towards understanding viral protein-induced changes in host metabolic flux. Our work here illustrates the power of employing CARS imaging and FLIM imaging of NAD(P)H fluorescence as complementary approaches to interrogate changes in metabolism.

## Materials and Methods

### Tissue Culture and Reagents

Human hepatoma cells (Huh7) were grown in DMEM medium supplemented with 100 nM nonessential amino acids, 50 U/mL penicillin, 50 µg/mL streptomycin, and 10% FBS (CANSERA, Rexdale, ON). The JFH-1 strain Core D2-GFP expression plasmids were previously described [14]. The GFP expression plasmid used in this study is the pIRES2-EGFP (Clontech, Mountainview, CA).

### Transfections

Huh7 cells were seeded at 1.0×10^5^ cells/well in borosilicate Lab-Tek chambers (VWR, Mississauga, ON). After 24 h, cells at 60–70% confluency were transfected with GFP-D2 or GFP expressing plasmid vector suspended in transfection media including lipofectamine 2000 (Invitrogen Canada Inc., Burlington, ON). After 4 h, DMEM with 20% FBS was added in equal volume to the chambers. Imaging was conducted 20 h post-transfection.

### qRT-PCR

For PPAR-α mRNA and 18S rRNA levels, 500 ng of total RNA was used for cDNA synthesis using the Superscript II kit (Invitrogen, Burlington, ON) according to the manufacturer’s protocol. Quantitative PCR (qPCR) was subsequently performed on an iCycler (Bio-Rad, Hercules, CA) using iQ SYBR Green Supermix (Bio-Rad, Hercules, CA), as per manufacturer’s protocol. Primer sequences are listed in Table S1. A 20 µL reaction was assembled according to the manufacturer’s protocol. For data analysis, the 2^−ΔΔCt^ method was used [69] using 18S rRNA levels as a reference gene. Mean fold changes in expression are shown relative to mock transfected samples.

### CARS and TPF Microscopies

The CARS microscopy procedure has been previously described [70]. Briefly, the CARS microscopy system uses a single femtosecond Ti:sapphire oscillator (Coherent Mira 900 modified to operate at 80 MHz) as the excitation source. The frequency difference between two input lasers, stokes and pump beam is equal to that of the Raman resonance of interest. The second longer wavelength (Stokes beam) is generated through use of a photonic crystal fiber (PCF), which produces power in the wavelength range of 1035 nm with negligible amplitude fluctuations. When overlapped with the 800 nm (pump beam) from the Ti:sapphire laser, this corresponds to the 2850 cm^−1^ Raman resonance of the C–H stretch. A modified Olympus Fluoview 300 laser scanning system and IX71 inverted microscope was used to carry out all CARS and two-photon imaging. A 40X 1.15 NA UAPO water immersion lens with a cover slip collection was used as the objective and the 0.55 NA long working distance condenser lens for collection in the forward direction. Light was directed to photomultiplier tubes (PMT) with enhanced red sensitivity (Hamamatsu R3896) and operated at a gain of about 530 V. Imaging was completed when the combined average powers reached approximately 120 mW for the pump and the Stokes outside the scan box. Total power transfer to the sample is less than 20%. Live cell samples were imaged in 4.2 cm^2^ Lab-Tek Chambers Slide System (NUNC, Rochester, NY). Optical sectioning of lipid droplets were imaged at 1 µm z-slices for a total z-stack analysis ranging from 7–12 µm depending on thickness of cell sample. Two photon fluorescence, obtained simultaneously from the same pump pulse, was detected using a separate detection channel using the Olympus Fluoview 300 laser scanning system and IX71 inverted microscope.

### TP-FLIM of NAD(P)H

For NADH FLIM, the center wavelength of the laser was tuned to 740 nm and had a full width half maximum (FWHM) spectral width of 15 nm which supports pulses of 100 fs. Cells were maintained in an enclosed chamber at 37°C and 5% CO_2_. The two-photon autofluorescence signal from NAD(P)H were collected in the de-scanned mode of the microscope to the photon-counting photomultiplier tube (PicoQuant GmbH, Berlin, Germany). The signals from PMT and reference photodiode were combined with the time-correlated single-photon counting electronics board (TCSPC, PicoHarp300, PicoQuant GmbH, Berlin, Germany). Data collection and data analysis is done by a commercial software package (SymPhoTime, PicoQuant GmbH, Berlin, Germany). Photon arrival times are recorded by the electronics with respect to the laser excitation pulse, and is done by acquiring data in reverse START-STOP mode. The START pulse is generated by the PMT while the STOP or SYNC pulse is provided directly from the laser via an internal photodiode. The operation of the PicoHarp 300 board in time-tagged time-resolved (TTTR) mode allows for the full time-resolved analysis of collected autofluorescence signal. A 450 nm centered 40 nm full width band pass filter (Chroma, Bellows Falls, Vermont ) was inserted in the fluorescence emission path from NADH that is central peak at 450 nm. An additional short-pass filter (Semrock 680 SP) was used to further exclude the backscattered 740 nm excitation light. For cellular imaging, the average power used at the focal plane of objective lens was ∼3 to 5 mW, which was lower than the laser power causing two photon damage and optimal for the prevention of photobleaching [71], [72]. Five separate images of grouped cells were collected per dish, with minimum spacing between observed groups. The total number of images acquired in the control and D2 treatment experiments was the product of images per dishes (n = 5) and number of imaging sessions [i.e. n = 40 (5x8)]. All the images were taken at 256×256 pixels resolution with the acquisition time in the range of 700–900 sec for accumulating good enough photon count statistics at the given laser power for further data analysis.

### FLIM Data Analysis

Data was analyzed with the commercially available Symphotime software package (PicoQuant, Germany) via a mathematical convolution of a model function with the instrument response function (IRF) and fitting of the model function to the experimental data as has been described previously. To calculate the lifetime from the composite decays of NADH, we convolved an IRF, I_instr_, with a double-exponential model function. The measured full width at half maximum of the IRF was determined to be 400 ps.

To illustrate the spatial distributions of the two forms of NADH, we constructed an image from the ratio of the early arriving photon counts to the total counts using ImageJ (NIH, US). We chose the cut-off of the early counts to be twice the time constant associated with the short decay (or 0.9 ns). In this way the intensity has a contribution for 86% of the short decay time population and 29% or the 2.5 ns decay time population or about 4 fold enriched for the short-decay-time species. Pixels with a total less than 20 counts were set to zero.

### Wide-field Fluorescence and DIC Imaging

Standard wide-field imaging of fluorescence and differential interference contrast (DIC) was done to identify transformed cells on the background of non-transformed cells in the same fields. Standard DIC prisms and polarizers for the Olympus IX71 microscope were temporarily inserted into the transmission path and the optical path for imaging onto a monochrome camera (Mightex CXE-B013-U). Illumination was achieved with a high intensity LED centered around 490 nm with collimation lens using 485 bandpass filter for excitation and 535 nm 22 nm wide for emission and a dichroic mirror reflecting below 500 nm. It is important to note that we verified that, within fields of Huh7 cells expressing GFP alone, the overall NAD(P)H fluorescence lifetime, intensities, and spatial distribution are indistinguishable from non-GFP expressing cells in the same image.

### Statistical Analysis

Student’s unpaired t-test was used to analyze the data, and *P-*values less than 0.05 were deemed significant.

## Supporting Information

Figure S1 Domain 2 of HCV core protein induces lipid accumulation in Huh7 cells. Huh7 cells were fixed and imaged by CARS and TPF microscopy 24 hours post-transfection with D2-GFP fusion protein. (A)–(D) Simultaneous CARS and TPF image of Huh7 cells expressing D2-GFP. A different field of view is shown in addition to the one displayed in Figure 1. (A) Typical TPF image showing localization of D2-GFP (green); (B) CARS image of lipid droplets (red) in the same field of view; (C) Overlay of the CARS and TPF images; (D) Magnified image from the inset of (C). Representative images are shown of three biological replicates. Scale bar = 10 µm.
(TIF)

Figure S2 GFP expression does not alter cytosolic lipid morphology in Huh7 cells. Huh7 cells were fixed and imaged by CARS and TPF microscopy 24 hours post-transfection with GFP alone. (A)–(C) Simultaneous CARS and TPF image of Huh7 cells expressing D2-GFP. A different field of view is shown in addition to the one displayed in Figure 1. (A) Typical TPF image showing localization of D2-GFP (green); (B) CARS image of lipid droplets (red) in the same field of view; (C) Overlay of the CARS and TPF images. Representative images are shown of three biological replicates. Scale bar = 10 µm.
(TIF)

Figure S3 Domain 2 of HCV core protein downregulates PPAR- α expression. Huh7 cells were transfected with D2-GFP. 24 hours post-transfection, total RNA was isolated and qRT-PCR was performed to measure PPAR**-**α expression. Relative abundances were normalized by 18S rRNA levels. Error bars represent standard error of the mean (n = 3; *p<0.05).
(TIF)

Figure S4 Domain 2 of HCV core protein increases NAD(P)H fluorescence. Additional field of view showing cells transfected with D2-GFP. Cells were imaged 24 hours post-transfection with TPF for NAD(P)H fluorescence, DIC, and CCD camera for GFP fluorescence. Left panel: Grayscale images of NAD(P)H fluorescence intensity signals. Right panel: Overlay of DIC images and GFP fluorescence (red). Representative images are shown of two biological replicates. Scale bar = 10 µm.
(TIF)

Figure S5 Domain 2 of HCV core alters cellular distribution of NAD(P)H fluorescence. Huh7 cells were transfected with either GFP (A) or D2-GFP (B) and imaged 24 hours post-transfection. NAD(P)H fluorescence intensity profiles are shown in the bottom panel. (B). GFP-transfected and non-transfected cells exhibited distinct foci as highlighted in the fluorescence intensity profile (lower panel) of the orange line indicated in the NAD(P)H fluorescence image (upper panel). In GFP transfected cells, the fluorescence intensity profile demonstrate a decrease to near-background levels of signal in the cytoplasm (highlighted by red arrows) – suggestive of distinct foci of NAD(P)H. Conversely, in D2-GFP transfected cells, less drastic fluctuations in fluorescence signal were observed in the cytoplasm. Images are representative of two independent experiments (ROIs ≥20). (C) Coefficients of variance were calculated for signal along NAD(P)H fluorescence intensity profiles for GFP and D2-GFP transfected cells. Average values are shown. Error bars represent the standard error of the mean (n = 5; *p<0.05).
(TIF)

Figure S6 TP-FLIM setup. Schematic diagram of the time-resolved fluorescence lifetime imaging (FLIM) using TCSPC electronics. BS: Beam Splitter; P: Polarizer; λ/2: Half wave-plate; M: Mirror; DM: Dichroic Mirror; Obj.: Objective; S: Sample; F: Filter; PMT: Photomultiplier tube; TCSPC: Time correlated single photon counting. Specific details of the set-up are discussed in the Materials and Methods.
(TIF)

Figure S7 Typical bi-exponential model fitting of NAD(P)H fluorescence decay curve. (A) Standard fitted curve is shown with χ^2^ close to 1 and residuals showing no noticeable systematic variations. (B) Representative color-coded image of NAD(P)H lifetimes in Huh7 cells and (C) NAD(P)H fluorescence intensity. Scale bar = 10 µm.
(TIF)

Figure S8 Domain 2 of HCV core induces alterations in NAD(P)H microenvironment. Additional field of view showing cells transfected with D2-GFP. Cells were imaged 24 hours post-transfection with FLIM for NAD(P)H lifetimes, DIC, and CCD camera for GFP fluorescence. Left panel: Color-coded images of average NAD(P)H lifetimes. Right panel: Overlay of DIC images and GFP fluorescence (red). Representative images are shown of two biological replicates. Scale bar = 10 µm.
(TIF)

Table S1 (DOCX)

Text S1 Data Analysis.
(DOCX)
