# Supplementary figures and images for: Fluorescence Lifetime Imaging of Alterations to Cellular Metabolism by Domain 2 of the Hepatitis C Virus Core Protein
Source: PLoS One. 2013 Jun 24;8(6):e66738. doi: 10.1371/journal.pone.0066738 (PMC3691201; doi:10.1371/journal.pone.0066738)

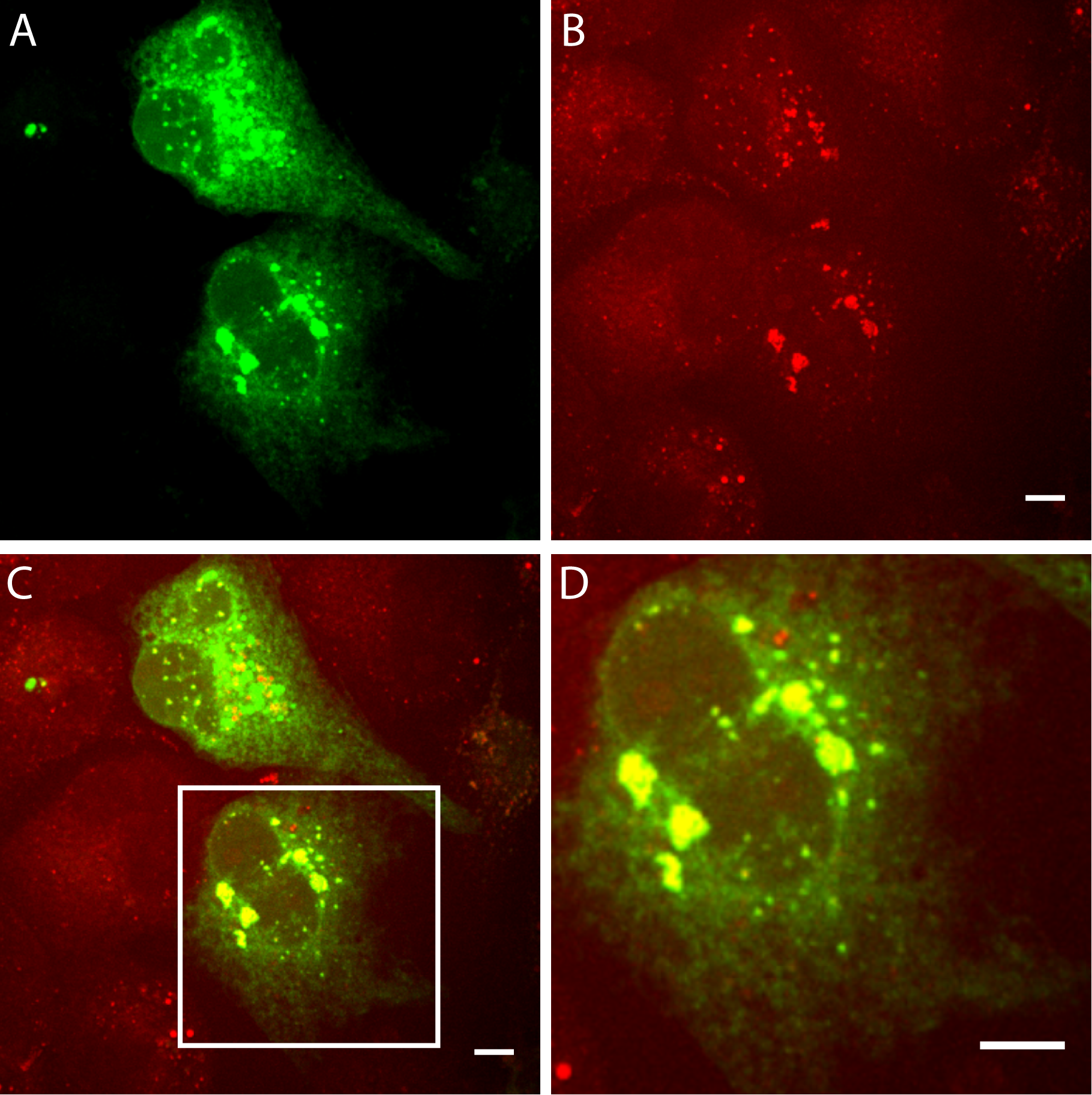

Supplement: Figure S1 — Domain 2 of HCV core protein induces lipid accumulation in Huh7 cells. Huh7 cells were fixed and imaged by CARS and TPF microscopy 24 hours post-transfection with D2-GFP fusion protein. (A)–(D) Simultaneous CARS and TPF image of Huh7 cells expressing D2-GFP. A different field of view is shown in addition to the one displayed in Figure 1. (A) Typical TPF image showing localization of D2-GFP (green); (B) CARS image of lipid droplets (red) in the same field of view; (C) Overlay of the CARS and TPF images; (D) Magnified image from the inset of (C). Representative images are shown of three biological replicates. Scale bar = 10 µm. (TIF) [file pone.0066738.s001.tif]

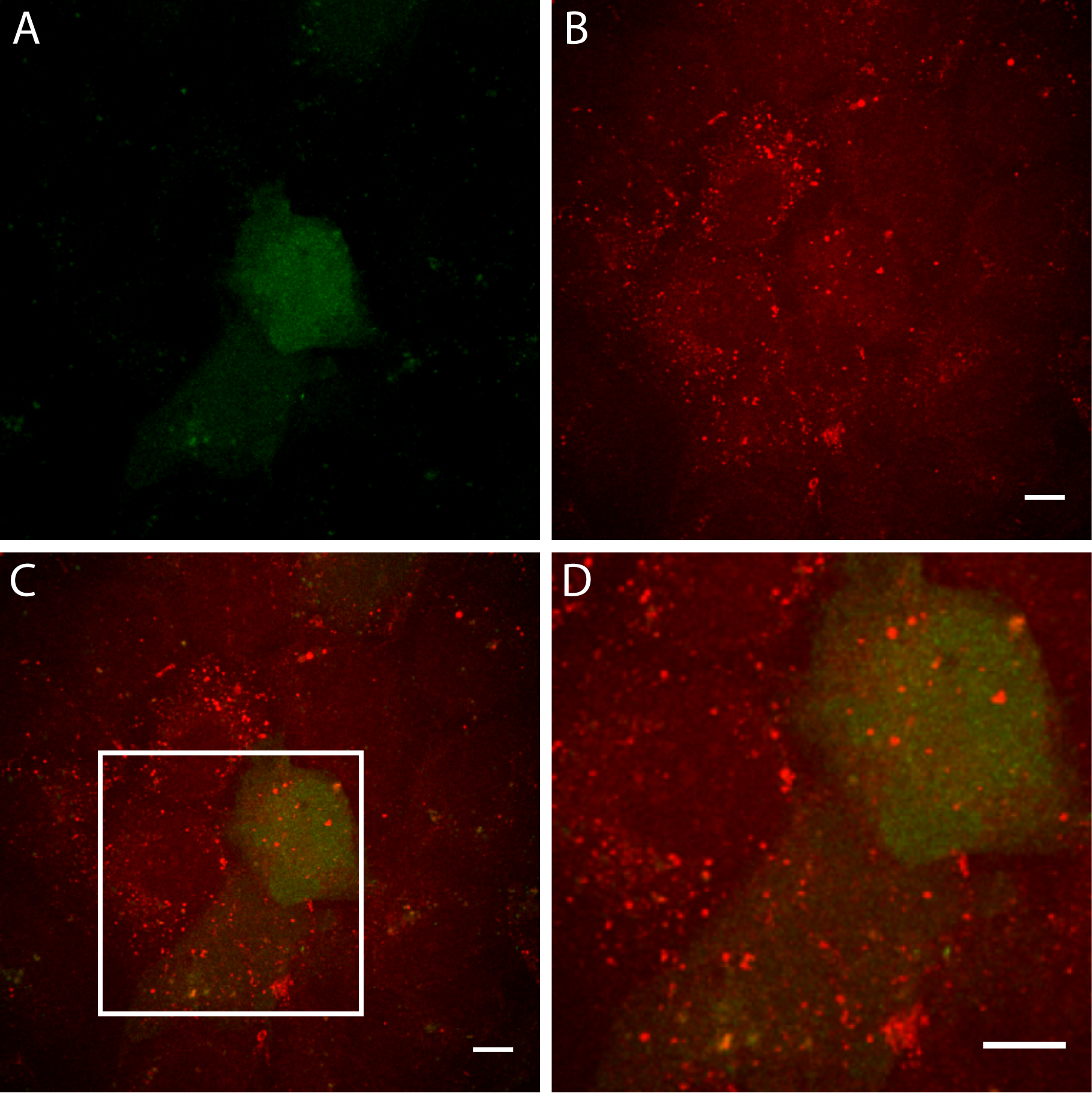

Supplement: Figure S2 — GFP expression does not alter cytosolic lipid morphology in Huh7 cells. Huh7 cells were fixed and imaged by CARS and TPF microscopy 24 hours post-transfection with GFP alone. (A)–(C) Simultaneous CARS and TPF image of Huh7 cells expressing D2-GFP. A different field of view is shown in addition to the one displayed in Figure 1. (A) Typical TPF image showing localization of D2-GFP (green); (B) CARS image of lipid droplets (red) in the same field of view; (C) Overlay of the CARS and TPF images. Representative images are shown of three biological replicates. Scale bar = 10 µm. (TIF) [file pone.0066738.s002.tif]

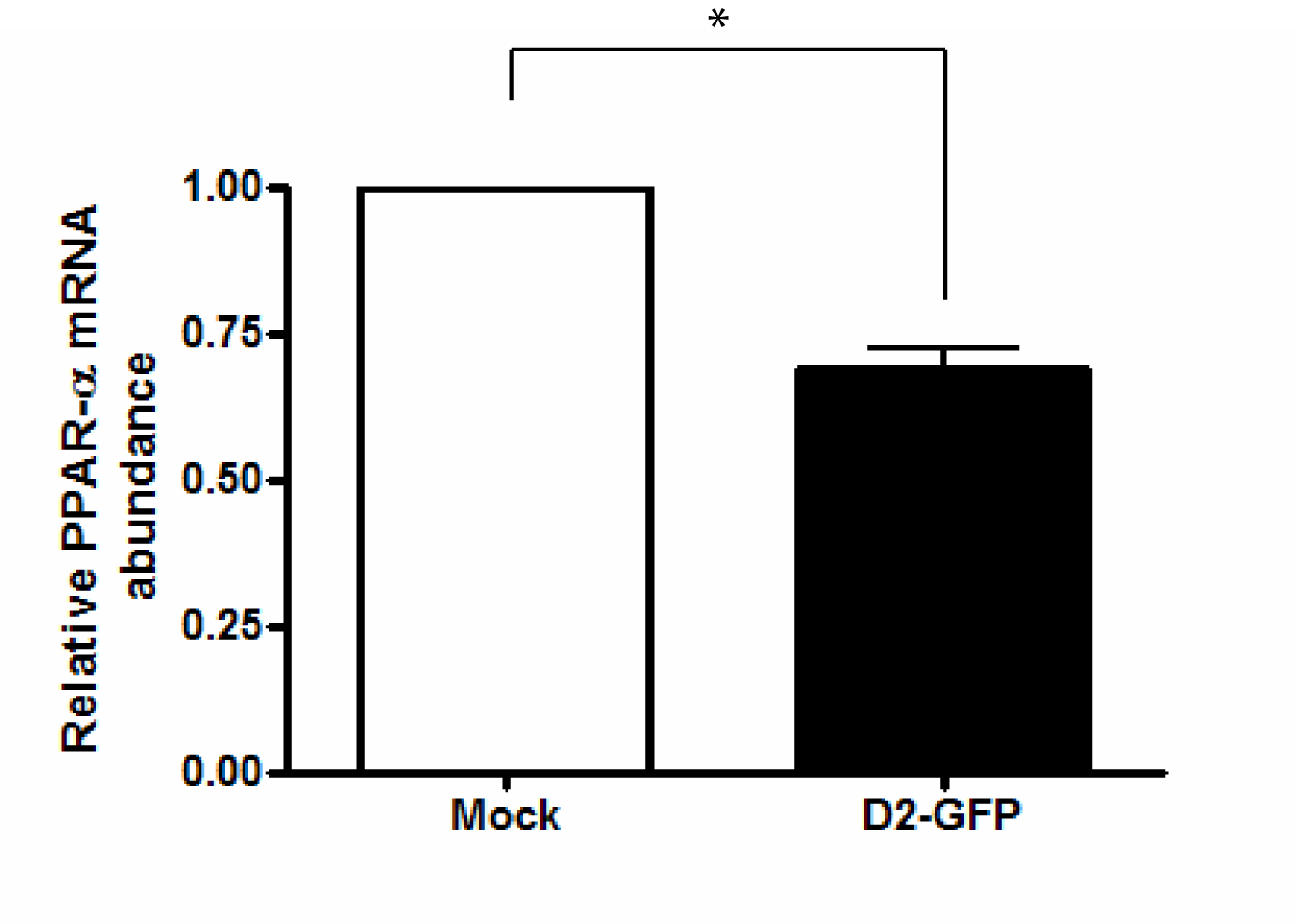

Supplement: Figure S3 — Domain 2 of HCV core protein downregulates PPAR- α expression. Huh7 cells were transfected with D2-GFP. 24 hours post-transfection, total RNA was isolated and qRT-PCR was performed to measure PPAR-α expression. Relative abundances were normalized by 18S rRNA levels. Error bars represent standard error of the mean (n = 3; *p<0.05). (TIF) [file pone.0066738.s003.tif]

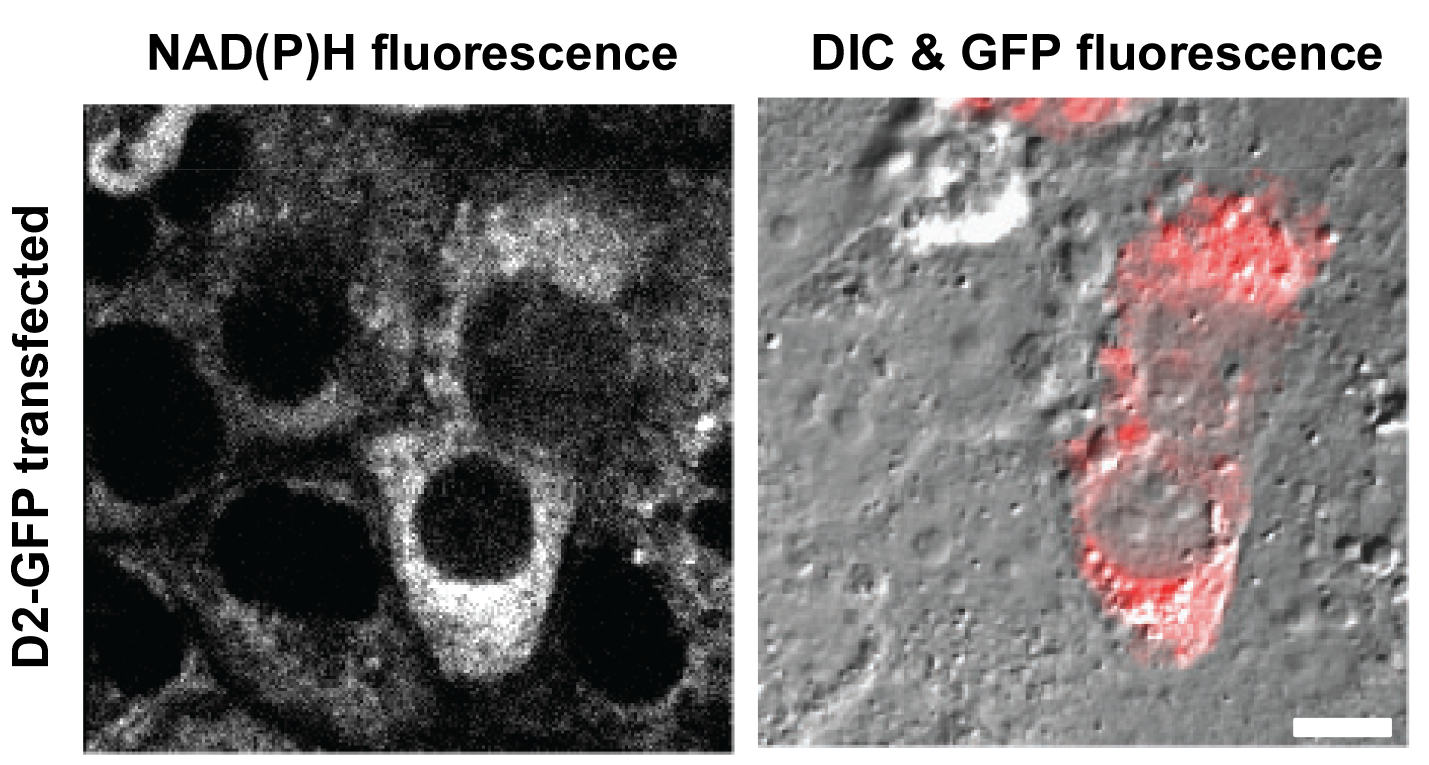

Supplement: Figure S4 — Domain 2 of HCV core protein increases NAD(P)H fluorescence. Additional field of view showing cells transfected with D2-GFP. Cells were imaged 24 hours post-transfection with TPF for NAD(P)H fluorescence, DIC, and CCD camera for GFP fluorescence. Left panel: Grayscale images of NAD(P)H fluorescence intensity signals. Right panel: Overlay of DIC images and GFP fluorescence (red). Representative images are shown of two biological replicates. Scale bar = 10 µm. (TIF) [file pone.0066738.s004.tif]

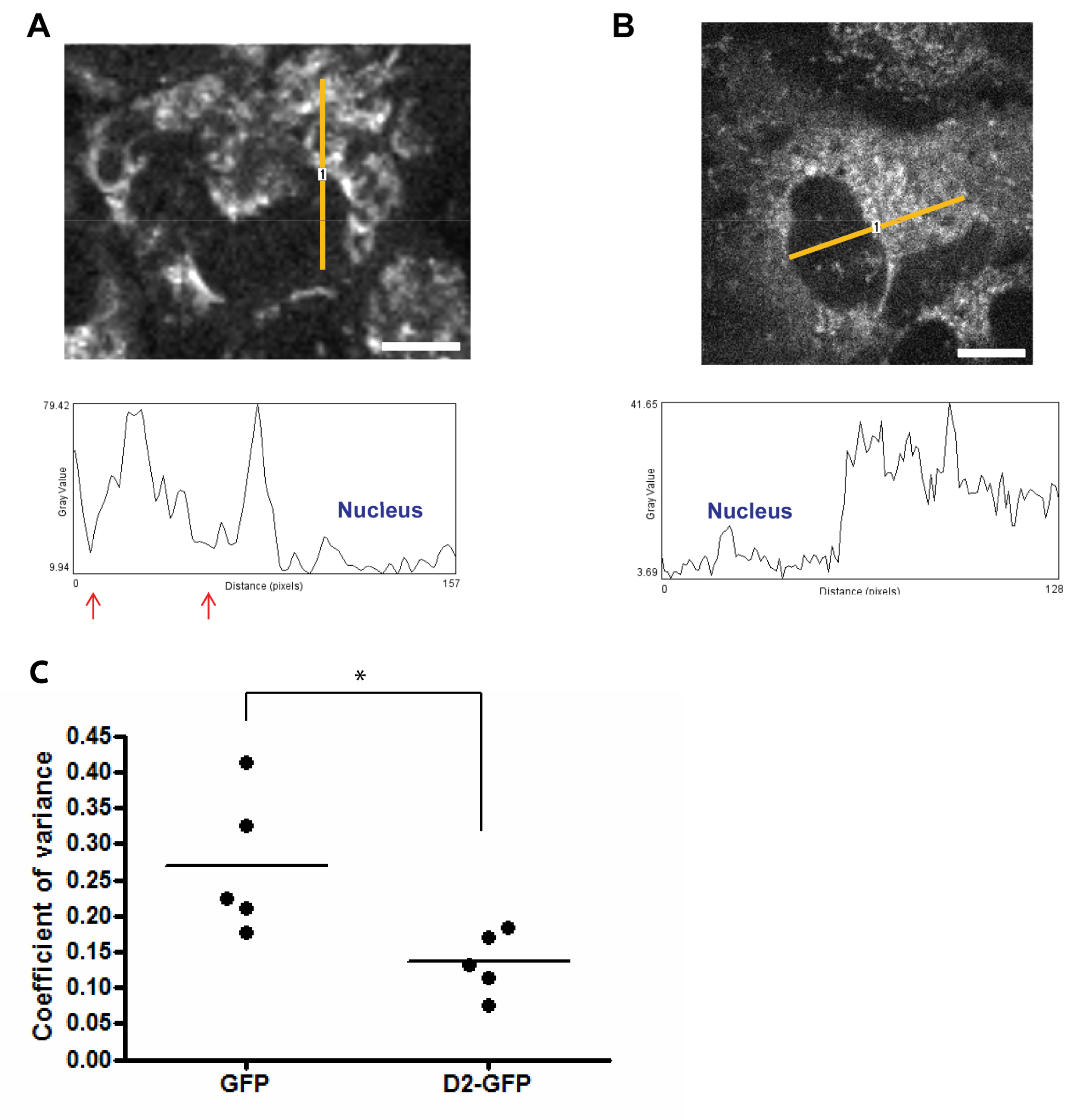

Supplement: Figure S5 — Domain 2 of HCV core alters cellular distribution of NAD(P)H fluorescence. Huh7 cells were transfected with either GFP (A) or D2-GFP (B) and imaged 24 hours post-transfection. NAD(P)H fluorescence intensity profiles are shown in the bottom panel. (B). GFP-transfected and non-transfected cells exhibited distinct foci as highlighted in the fluorescence intensity profile (lower panel) of the orange line indicated in the NAD(P)H fluorescence image (upper panel). In GFP transfected cells, the fluorescence intensity profile demonstrate a decrease to near-background levels of signal in the cytoplasm (highlighted by red arrows) – suggestive of distinct foci of NAD(P)H. Conversely, in D2-GFP transfected cells, less drastic fluctuations in fluorescence signal were observed in the cytoplasm. Images are representative of two independent experiments (ROIs ≥20). (C) Coefficients of variance were calculated for signal along NAD(P)H fluorescence intensity profiles for GFP and D2-GFP transfected cells. Average values are shown. Error bars represent the standard error of the mean (n = 5; *p<0.05). (TIF) [file pone.0066738.s005.tif]

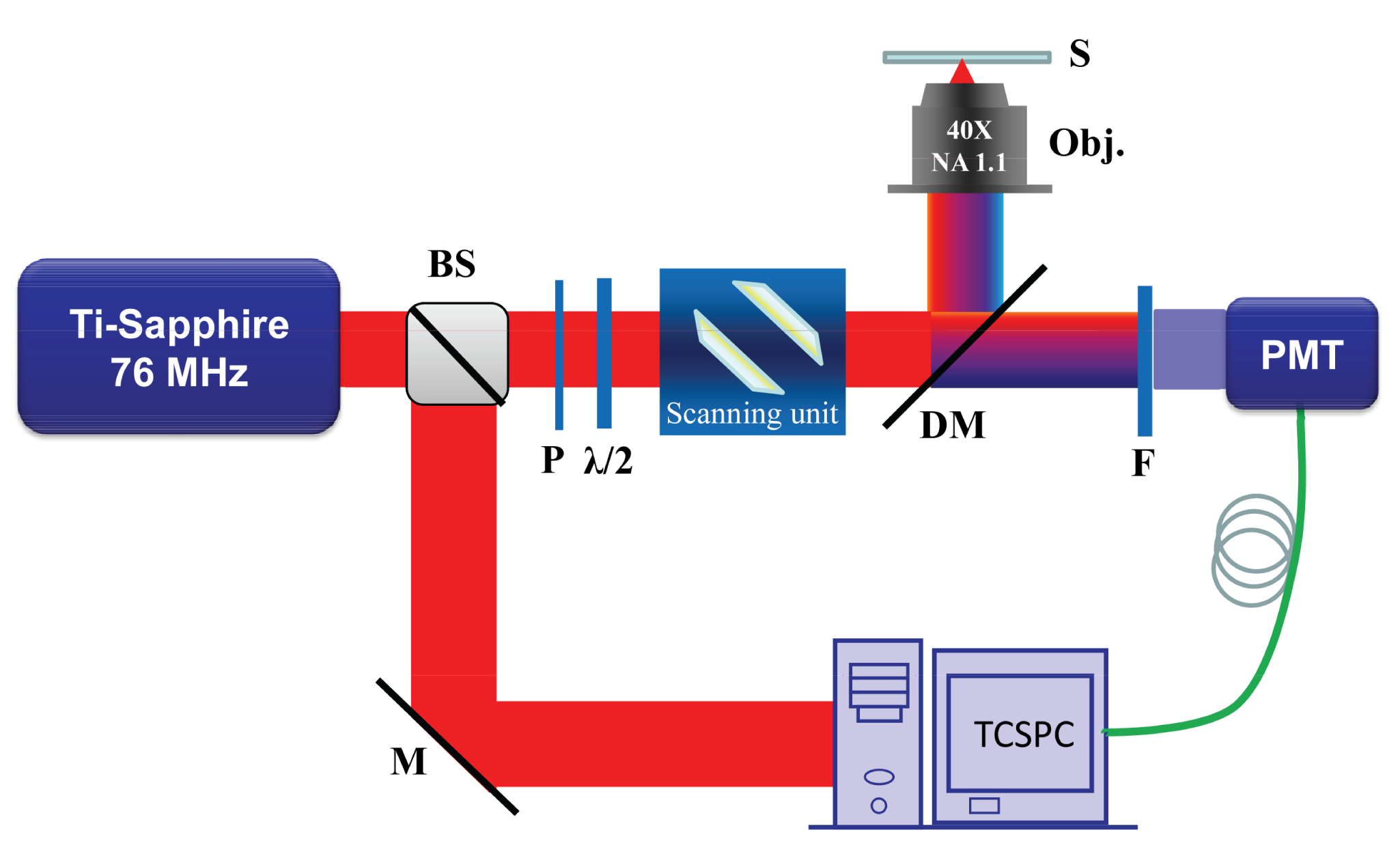

Supplement: Figure S6 — TP-FLIM setup. Schematic diagram of the time-resolved fluorescence lifetime imaging (FLIM) using TCSPC electronics. BS: Beam Splitter; P: Polarizer; λ/2: Half wave-plate; M: Mirror; DM: Dichroic Mirror; Obj.: Objective; S: Sample; F: Filter; PMT: Photomultiplier tube; TCSPC: Time correlated single photon counting. Specific details of the set-up are discussed in the Materials and Methods. (TIF) [file pone.0066738.s006.tif]

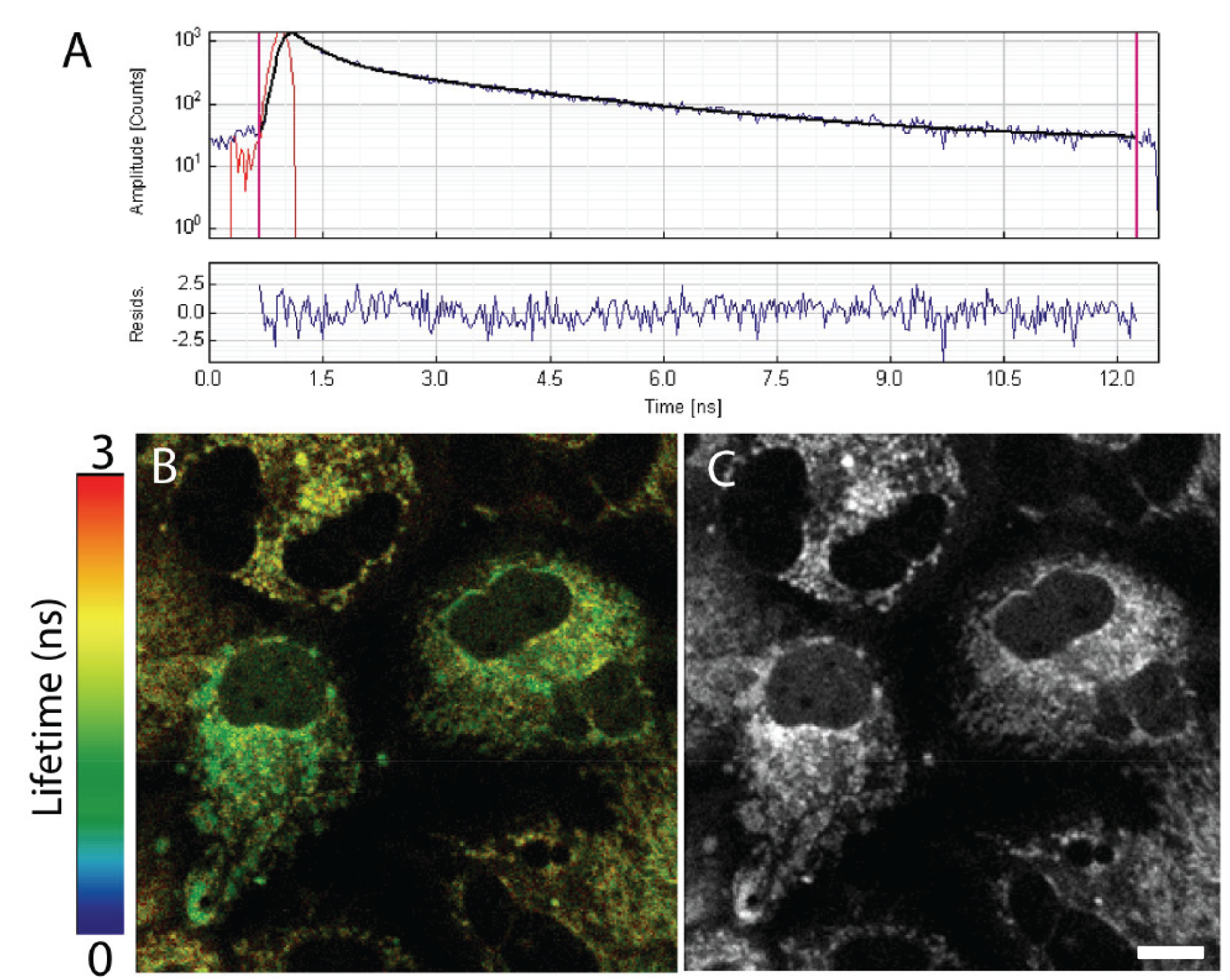

Supplement: Figure S7 — Typical bi-exponential model fitting of NAD(P)H fluorescence decay curve. (A) Standard fitted curve is shown with χ2 close to 1 and residuals showing no noticeable systematic variations. (B) Representative color-coded image of NAD(P)H lifetimes in Huh7 cells and (C) NAD(P)H fluorescence intensity. Scale bar = 10 µm. (TIF) [file pone.0066738.s007.tif]

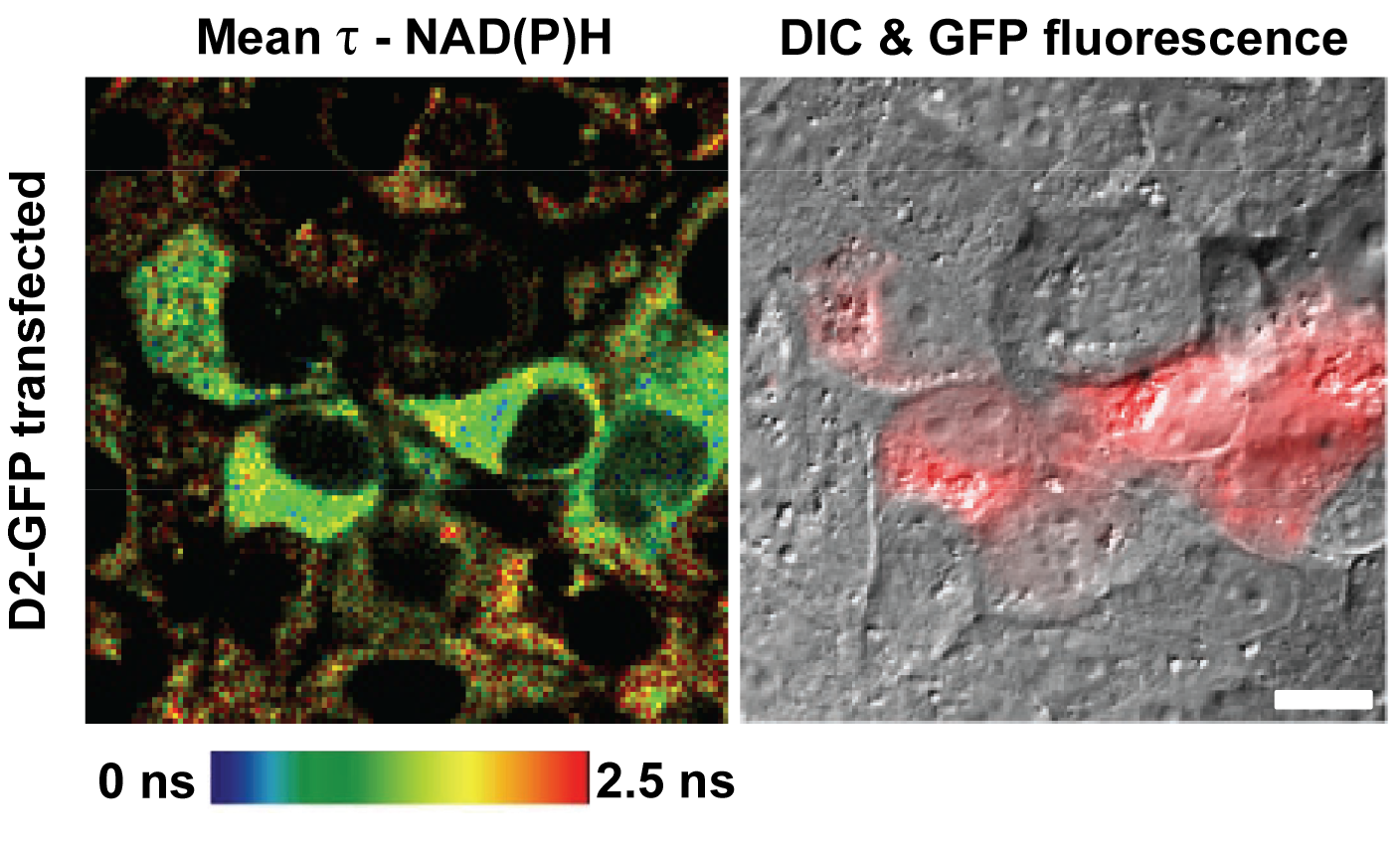

Supplement: Figure S8 — Domain 2 of HCV core induces alterations in NAD(P)H microenvironment. Additional field of view showing cells transfected with D2-GFP. Cells were imaged 24 hours post-transfection with FLIM for NAD(P)H lifetimes, DIC, and CCD camera for GFP fluorescence. Left panel: Color-coded images of average NAD(P)H lifetimes. Right panel: Overlay of DIC images and GFP fluorescence (red). Representative images are shown of two biological replicates. Scale bar = 10 µm. (TIF) [file pone.0066738.s008.tif]
