# Supplementary material for: Fluorescence Lifetime Imaging of Alterations to Cellular Metabolism by Domain 2 of the Hepatitis C Virus Core Protein
Source: PLoS One. 2013 Jun 24;8(6):e66738. doi: 10.1371/journal.pone.0066738 (PMC3691201; doi:10.1371/journal.pone.0066738)
Supplement: Table S1 — (DOCX) [file pone.0066738.s009.docx]

| Primer | Sequence |
| --- | --- |
| 18S rRNA FWD | GCGATGCGGCGGCGTTATTC |
| 18S rRNA REV | CAATCTGTCAATCCTGTCCGTGTCC |
| PPAR-α FWD | CTATCATTTGCTGTGGAGATCG |
| PPAR-α REV | AAGATATCGTCCGGGTGGTT |

**Table S1. qPCR Primers**
