# Supplementary material for: Fluorescence Lifetime Imaging of Alterations to Cellular Metabolism by Domain 2 of the Hepatitis C Virus Core Protein
Source: PLoS One. 2013 Jun 24;8(6):e66738. doi: 10.1371/journal.pone.0066738 (PMC3691201; doi:10.1371/journal.pone.0066738)
Supplement: Text S1 — Data Analysis. (DOCX) [file pone.0066738.s010.docx]

**Text S1. Data Analysis**

We wished to estimate the relative increase in population of NAD(P)H [both free (1) and bound (2)]. The amplitude (a_i_) in a FLIM image is proportional to the system detection efficiency (φ), two photon cross-section (σ), laser intensity (I), radiative lifetime (τ_radiative_), and species concentration (c_i_) [1]:

$a_{i}=\frac{\varphi\sigma I^{2}}{\tau_{radiative}}c_{i}$ (1)

Assuming negligible differences in photon cross-section, laser intensity, and radiative lifetime (which is dependent on the refractive index), we can estimate the increase in NAD(P)H concentration in D2-expressing cells relative to non-transfected cells as follows:

$\begin{matrix} \frac{(c_{1}+c_{2})_{D2}}{(c_{1}+c_{2})_{Mock}}=\frac{(a_{1}+a_{2})_{D2}}{(a_{1}+a_{2})_{Mock}} \\ =\frac{\left(a_{2}\right)_{D2}}{\left(a_{2}\right)_{Mock}}\frac{\left(\frac{a_{1-D2}}{a_{2-D2}}+1\right)}{\left(\frac{a_{1-Mock}}{a_{2-Mock}}+1\right)} \end{matrix}$ (2)

For the $\frac{\left(a_{2}\right)_{D2}}{\left(a_{2}\right)_{Mock}}$ ratio, we simply rearrange the equation relating total detected fluorescence to measured amplitudes and lifetimes (τ_i_) [1]:

$Fluorescence_{i}=\sum_{i} a_{i}\tau_{i}$ (3)

$\begin{matrix} \frac{Fluorescence_{D2}}{Fluorescence_{Mock}}=\frac{(a_{1}\tau_{1}+a_{2}\tau_{2})_{D2}}{(a_{1}\tau_{1}+a_{2}\tau_{2})_{Mock}} \\ =\frac{\left(a_{2}\right)_{D2}}{\left(a_{2}\right)_{Mock}}\frac{\left(\frac{a_{1}}{a_{2}}\tau_{1}+\tau_{2}\right)_{D2}}{\left(\frac{a_{1}}{a_{2}}\tau_{1}+\tau_{2}\right)_{Mock}} \end{matrix}$ (4)

Combining equations (2) and (4) enables an approximation of relative NAD(P)H populations.

**References**

1. Chia TH, Williamson A, Spencer DD, Levene MJ (2008) Multiphoton fluorescence lifetime imaging of intrinsic fluorescence in human and rat brain tissue reveals spatially distinct NADH binding. Opt Express 16: 4237-4249
